# Acceptance du vaccin anti-COVID-19 au Maroc: étude transversale auprès des étudiants

**DOI:** 10.11604/pamj.2021.38.381.27748

**Published:** 2021-04-19

**Authors:** Yassine Samouh, Mohamed Réda Sefrioui, Sanae Derfoufi, Adnane Benmoussa

**Affiliations:** 1 Laboratoire des Sciences des Médicaments, Recherche Biomédicale et Biotechnologique, Faculté de Médecine et de Pharmacie, Université Hassan II, Casablanca, Maroc

**Keywords:** Acceptance du vaccin, vaccin anti-COVID-19, les étudiants, perception du risque, Maroc

## Aux éditeurs du Pan African Medical Journal

La pandémie de COVID-19 s'est déployée à travers le monde entier en engendrant un impact négatif sur divers aspects de la société. Une des stratégies les plus prometteuses pour réduire le virus, et sauver des vies, c´est de développer un vaccin efficace et sûr. Les chercheurs du monde entier ont travaillé en collaboration pour atteindre cet objectif avec plusieurs vaccins candidats testés [1]. Cependant, le succès du programme de vaccination contre la COVID-19 dépendra en grande partie de l´acceptance du vaccin par la population.

Au Maroc, les étudiants universitaires ont repris les cours présentiels après l´été, et la plupart d'entre eux vivent avec des colocataires, et ils doivent se déplacer entre leur domicile et leur faculté. Les étudiants pourraient être alors l'une des populations cibles de la vaccination contre la COVID-19, et pourraient être aussi considérés comme une population appropriée pour étudier leurs attitudes d'accepter une nouvelle vaccination parce qu'ils sont ouverts d'esprit, éduqués et censés réagir rapidement aux problèmes de santé publique. Il nous a paru intéressant d´explorer les attitudes à l'égard d'un futur vaccin anti-COVID-19, d´étudier les facteurs prédicteurs de l'acceptance de se faire vacciner contre la COVID-19 chez les étudiants. Ce qui contribuera au développement de stratégies efficaces pour promouvoir l'adoption du vaccin anti-COVID-19 au sein de cette population. Nous avons mené une étude transversale basée sur un questionnaire auto-administré chez les étudiants, entre le 14 novembre et le 28 novembre 2020. Une invitation par e-mail comprenait un lien Web du questionnaire, les objectifs de l'étude, les informations sur l´anonymat et la confidentialité des données, a été envoyée aux étudiants. La fiche d´exploitation comportait: (1) les caractéristiques sociodémographiques, (2) l´intention de se faire vacciner, (3) les barrières qui empêchent les hésitants et les réticents de se faire vacciner contre la COVID-19, (4) l´exposition personnelle à contracter la COVID-19, (5) la peur de la maladie COVID-19, (6) la vulnérabilité perçue, (7) la criticité perçue de la maladie COVID-19. Toutes les données obtenues étaient saisies dans le programme SPSS [version 22]. Une Corrélation de Spearman a été utilisée pour déterminer les facteurs associés à l´intention de se faire vacciner contre la COVID-19 chez les étudiants.

**Figure 1:**
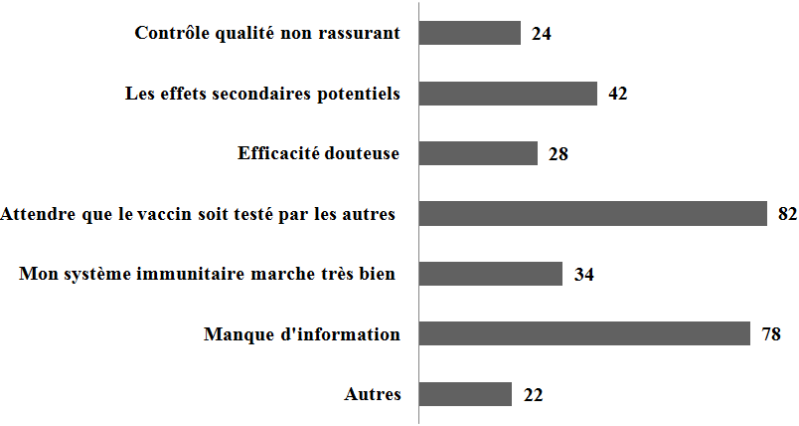
les barrières qui empêchent les participants réticents/hésitants à prendre le vaccin anti-COVID-19

Au total, 870 étudiants ont participés à cette enquête. Les caractéristiques démographiques sont présentées dans le Tableau 1. L'analyse descriptive des 870 étudiants qui ont répondu à la question sur l'intention de se faire vacciner a montré que 560 étudiants (64,4%) ont déclaré qu'ils choisiraient certainement ou probablement de se faire vacciner contre le COVID-19; de l'autre côté, 310 étudiants (35,6%) ont déclaré qu'ils ne se feraient pas ou ne seraient pas sûrs de se faire vacciner. Nous avons demandé aux répondants réticents et hésitants de nous déclarer les principales barrières qui leur empêchent de se faire vacciner contre la COVID-19 (Figure 1). L'analyse statistique n'a montré aucune corrélation entre le sexe, l´exposition personnelle à contracter la maladie COVID-19 et l'acceptance du vaccin. L’analyse statistique a montré une corrélation entre le niveau d´acceptance de se faire vacciner contre le coronavirus COVID-19 et la peur de la maladie COVID-19 et la gravité perçue de la maladie COVID-19. De plus, lors de l'analyse comparant les étudiants suivant des études sanitaires et les étudiants suivant d´autres domaines d´étude, nous n'avons trouvé aucune différence significative en matière de l'acceptance du vaccin anti-COVID-19.

**Tableau 1:** les caractéristiques de la population d’étude (N=870)

	Variables	Pourcentage (%)
**Age (ans)**	Moyenne = 22,9	Écart-type = ±2
**Sexe**	Homme	42%
	Femme	58%
**Domaine d'étude**	Domaine de la santé	47,3%
	Autres domaines	52,7%
**Niveau d'instruction**	Bac plus	
	1 an	10,6%
	2 ans	13%
	3 ans	14,5%
	4 ou 5 ans	32,7%
	Plus de 5 ans	29,2%
	Oui, certainement	35,3%
	Oui, Probablement	29,1%
**L'intention de se faire vacciner**	Neutre/sans opinion	20,5%
	Plutôt non	7,1%
	Non, certainement	8%

Le taux d´acceptance du vaccin anti-COVID-19 trouvé chez les étudiants lors d´une étude transversale réalisée en Italie, était de 86.1% [2]. Alors que seulement 64,4% des étudiants ont déclaré qu'ils choisiraient de se faire vacciner dans notre étude. Cela signifie que dans notre échantillon, plus de 3 étudiants sur 10 montraient une faible intention de se faire vacciner. Nos résultats montraient qu'une gravité perçue et une peur du COVID-19 plus élevées étaient positivement associées à une acceptance plus élevée du vaccin. La gravité perçue du COVID-19 pourrait augmenter la volonté de se faire vacciner contre la COVID-19 dans tous les groupes d'âge [3]. Pour les étudiants, la peur de l'infection peut être causée non seulement par des résultats cliniques potentiels, mais aussi par des conséquences sociales telles que l'isolement social et la stigmatisation de leurs pairs.

Contrairement au résultat énoncé par Graffigna *et al*. [4] en étudiant l'acceptance du vaccin anti-COVID-19, nous avons constaté dans notre étude que la vulnérabilité perçue face au COVID-19 n'était pas significativement associée à l'acceptance du vaccin chez les étudiants. Cela peut être attribué au biais optimiste chez certains étudiants qui ne considèrent pas le virus comme une menace sérieuse. En comparant les étudiants suivant des cursus en domaines de la santé avec ceux qui suivaient les autres domaines d´études, nous n'avons trouvé aucune différence significative en termes d´acceptance du vaccin anti-COVID-19 (p = 0,27). Ce constat suggère que l'attitude vaccinale n'est pas seulement influencée par le niveau de connaissances des étudiants en matière de santé, mais probablement par d'autres facteurs motivationnels et psychologiques, y compris le bon sens quant à la valeur de la vie civique et de la solidarité sociale [5].

Les inquiétudes concernant le futur vaccin anti-COVID-19 peuvent constituer des cibles importantes pour d'éventuels programmes éducatifs interventionnels visant à améliorer les taux de vaccination [5]. Une approche multidisciplinaire de l´enseignement pourrait contribuer à dépasser l´approche traditionnelle de l´information sanitaire. Enseigner les questions liées à la santé publique grâce à un programme éducatif approprié dans plusieurs disciplines, y compris les sciences sociales, psychologiques et comportementales, demeure la clé pour mieux éduquer les étudiants sur les barrières à la demande de service de vaccination [6].

Conclusion: cette étude fournit des données préliminaires pour comprendre l'acceptance du vaccin anti-COVID-19 chez les étudiants. En tant que communauté scientifique, nous devons agir pour éduquer, informer et intervenir pour augmenter les taux d´acceptance du vaccin anti-COVID-19 dans l'ensemble de la population.
